# Evaluation of the Impact of Osteoporosis and Rheumatoid Arthritis on the Outcomes of Nonsurgical Endodontic Therapy: A Prospective Cohort Study

**DOI:** 10.7759/cureus.116950

**Published:** 2026-09-25

**Authors:** Ajay Chhabra, Sona J Parvathy, Himani Mehra, Amit Kumar, Shilpa R, Arpita Banerjee, Vandana Chabbra, Bhupendra Ramesh Babaria

**Affiliations:** 1 Department of Conservative Dentistry and Endodontics, All India Institute of Medical Sciences, Kalyani, IND; 2 Department of Orthopedics, All India Institute of Medical Sciences, Kalyani, IND; 3 Department of Dentistry, All India Institute of Medical Sciences, Kalyani, IND; 4 Department of Oral and Maxillofacial Surgery, Dr. Harvansh Singh Judge Institute of Dental Sciences and Hospital, Chandigarh, IND

**Keywords:** cone-beam computed tomography, nonsurgical endodontic therapy, osteoporosis, periapical healing, periapical index, rheumatoid arthritis

## Abstract

Introduction: Osteoporosis (OP) and rheumatoid arthritis (RA) can potentially disrupt periapical healing following nonsurgical endodontic therapy (NSET) due to changes in bone metabolism and persistent inflammation.

Methods: We assigned 80 participants to three groups: healthy controls (n = 40), OP (n = 20), and RA (n = 20). All participants received standardized NSET. We assessed periapical healing using digital radiovisiography and cone-beam computed tomography scans at baseline and one, three, and six months, based on measurements of periapical radiolucency and periapical index (PAI) scores. Data were analyzed using Wilcoxon signed-rank and Kruskal-Wallis tests (p < 0.05).

Results: There was statistically significant improvement in radiographic healing among all groups during the six-month study period (p < 0.001). PAI scores at baseline were similar among all groups. Healthy participants had significantly more periapical healing compared with OP and RA groups at six months (p < 0.05). Higher mean values of residual radiolucency and PAI scores were noted among the OP and RA groups.

Conclusions: OP and RA groups exhibited delayed periapical healing after NSET when compared with healthy controls; however, all groups experienced significant periapical healing following therapy.

## Introduction

Apical periodontitis results from an inflammatory reaction of the periapical tissues to infection of the root canal system. Apical periodontitis occurs when endodontic pathogens interact with host defenses, resulting in periapical bone destruction and soft tissue inflammation. The goal of nonsurgical endodontic therapy (NSET) is to disinfect the root canal space and allow periapical tissues to heal [1]. Although treatment outcomes are predictable, successful periapical healing requires both proper disinfection and obturation of the root canal system as well as adequate host immune regulation and bone regeneration capacities [2]. Contemporary endodontic approaches emphasize effective root-canal preparation and the management of anatomically complex canals to improve the safety, efficiency, and predictability of treatment outcomes [3]. Factors influencing NSET outcomes include microbial, procedural, tooth-related, and patient-related factors. Recent studies highlight systemic health, a patient-related factor, as influential on periapical healing postendodontic treatment. Chronic systemic diseases can alter immune and inflammatory responses and bone remodeling, potentially affecting healing after NSET [4]. While local factors are typically the primary determinants of successful outcomes, systemic health can significantly modify endodontic treatment results [5]. Osteoporosis (OP) and rheumatoid arthritis (RA) are chronic systemic diseases that affect bone metabolism while sustaining inflammation. OP is defined by low bone mineral density and compromised bone microarchitecture, while RA is an autoimmune disease characterized by inflammation-mediated bone destruction [6]. Both diseases could negatively affect bone remodeling and repair mechanisms, hindering the healing process of periapical lesions after NSET [7]. Previous studies have investigated diabetes mellitus' effect on endodontic treatment outcomes extensively. However, limited evidence exists concerning OP and RA influence on periapical healing after apical surgery. Published articles on this topic are largely retrospective and/or focused on the prevalence of apical periodontitis, instead of posttreatment healing. There is a lack of prospective clinical studies that incorporate standardized treatment methods with volumetric radiographic measurements [8].

Therefore, this prospective observational study aimed to evaluate the impact of OP and RA on periapical healing following NSET using serial radiographic assessment with radiovisiography (RVG) and cone-beam computed tomography (CBCT). By simultaneously evaluating two systemic conditions that alter bone metabolism and inflammatory pathways under a standardized treatment protocol, this study provides a comprehensive assessment of their potential influence on endodontic treatment outcomes.

## Materials and methods

Study design and setting

This prospective observational cohort study was undertaken in the Department of Dentistry between June 24, 2024, and April 24, 2026. Ethical approval was granted by the Institutional Ethics Committee (IEC/AIIMS/Kalyani/certificate/2024/099), and the study was performed in accordance with the Declaration of Helsinki and reported in compliance with the STrengthening the Reporting of OBservational studies in Epidemiology guidelines. Participants were enrolled after providing written informed consent.

Study population and group allocation

A study cohort of 80 patients requiring NSET were enrolled into three mutually exclusive groups: healthy controls (n = 40), OP (n = 20), and RA (n = 20). Participants were classified according to their established systemic diagnosis, and individuals with other systemic diseases were excluded. Patients with coexisting OP and RA were not included in either disease-specific group to maintain distinct exposure categories for comparative analysis.

Eligibility criteria

The control group comprised systemically healthy participants without a documented diagnosis of OP, RA, or other systemic disease meeting the exclusion criteria. Participants with systemic conditions that could substantially influence bone metabolism or periapical healing were excluded according to the predefined eligibility criteria.

Participants aged >18 years with mature permanent posterior teeth diagnosed with pulpal necrosis and radiographic evidence of apical periodontitis were included. OP was confirmed by dual-energy X-ray absorptiometry using the established diagnostic criterion of a T-score less than or equal to -2.5. RA was diagnosed according to the 2010 American College of Rheumatology/European Alliance of Associations for Rheumatology classification criteria (score ≥6) [9]. Exclusion criteria included retreatment cases, teeth without periapical radiolucency, immature teeth, internal or external resorption, root perforation, vertical root fracture, periodontally compromised teeth, anterior or deciduous teeth, other systemic diseases, and patients unwilling to participate.

Sample size calculation

The required sample size was estimated a priori based on an anticipated healing rate of 80% in systemically healthy participants and 40% in participants with systemic disease. Assuming a two-sided significance level (α) of 0.05, 80% statistical power (1 - β = 0.80), and a 10% anticipated attrition rate, the required sample size was determined to provide adequate power for the primary comparative assessment of periapical healing between the study groups. The final sample comprised 80 participants, including 40 healthy controls, 20 participants with OP, and 20 participants with RA.

Endodontic treatment protocol

All procedures were done by a single operator under 3× magnification. After administration of 2% lidocaine with 1:80,000 epinephrine (ICPA Xicaine, India) and rubber dam isolation (Coltene/Whaledent Inc., Cuyahoga Falls, OH), coronal access was established using an EndoAccess bur (Dentsply Sirona, Germany), and deroofing and refinement of the pulp chamber were carried out using an Endo-Z bur (Dentsply Sirona, Germany). Canals were negotiated with #10 or #15 K-files (MANI Inc., Japan). We established working length using an electronic apex locator (Root ZX, J. Morita, Japan) and confirmed it radiographically with RVG. The definitive working length was standardized at 0.5-1 mm short of the radiographic apex to minimize overinstrumentation and ensure appropriate apical termination.

ProTaper Gold rotary system (Dentsply Maillefer, Switzerland) was used for root canal preparation, following the crown-down technique. Throughout instrumentation, a standardized irrigation protocol was followed in accordance with the 2024 evidence-based guidelines [10]. Each canal was irrigated with 5 mL of 3% sodium hypochlorite (NaOCl) (Prime Dental Sodium Hypochlorite, India) after the use of each file using a 30-gauge side-vented needle positioned 1-2 mm short of the working length. RC-Prep, an ethylenediaminetetraacetic acid (EDTA)-based lubricant (Waldent Innovations Pvt. Ltd., India), was used during instrumentation. Final irrigation consisted of 5 mL of 3% NaOCl for one minute, followed by 5 mL of 17% EDTA (SafeEndo Smart Prep Liquid Pvt. Ltd., India) for one minute, and a final rinse with 3% sodium hypochlorite for 30 seconds. Sterile saline was used between irrigants to prevent chemical interactions.

Root canals were obturated in the same visit using gutta-percha and Seal-Pex epoxy resin-based sealer (Waldent Innovations Pvt. Ltd., India) by the lateral condensation technique, followed by permanent composite restoration.

Radiographic assessment and follow-up

Serial CBCT examinations were incorporated to permit standardized three-dimensional assessment of changes in the periapical lesion during the predefined follow-up period. The imaging schedule was established prospectively as part of the study protocol and was performed using the lowest practical exposure parameters consistent with adequate diagnostic image quality. The potential diagnostic benefit of serial three-dimensional assessment was considered against radiation exposure, and imaging was restricted to the study participants and time points specified in the approved research protocol.

Outcome measures

Clinical and radiographic evaluations were performed at baseline and after one, three, and six months, as shown in Figures 1-3. Assessment of periapical healing was done using RVG and CBCT. The primary outcome was change in CBCT-based Periapical Index (CBCT-PAI) scores according to Estrela et al. [11], together with changes in periapical radiolucency measurements.

**Figure 1:**
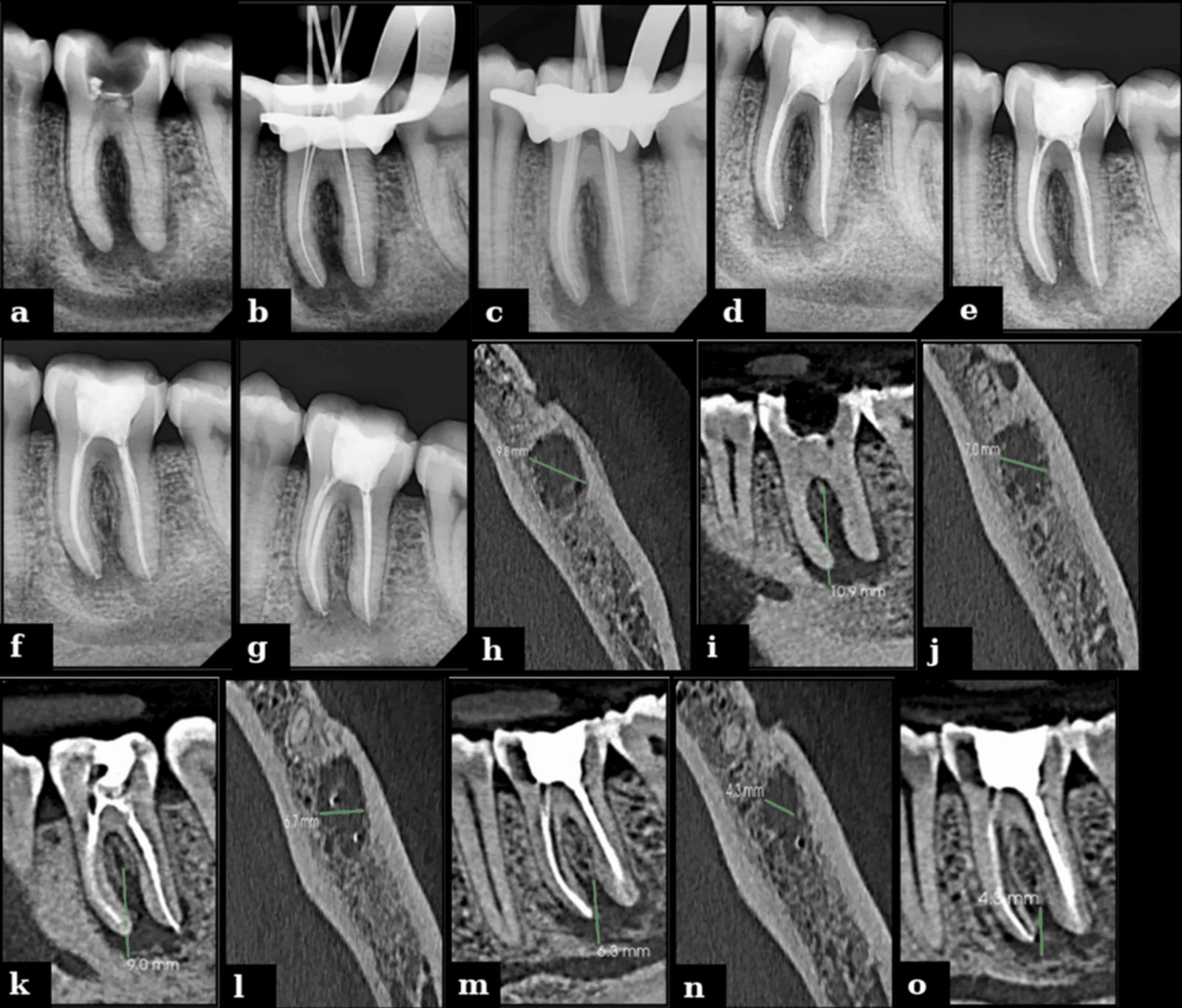
Serial radiographic evaluation of the control group Two-dimensional RVG: (a) preoperative view, (b) working-length determination, (c) master cone, (d) obturation and postendodontic restoration, (e) one-month follow-up, (f) three-month follow-up, and (g) six-month follow-up. Three-dimensional CBCT, paired axial and sagittal views: (h,i) preoperative, (j,k) one-month follow-up, (l,m) three-month follow-up, and (n,o) six-month follow-up CBCT: cone-beam computed tomography; RVG: radiovisiography

**Figure 2:**
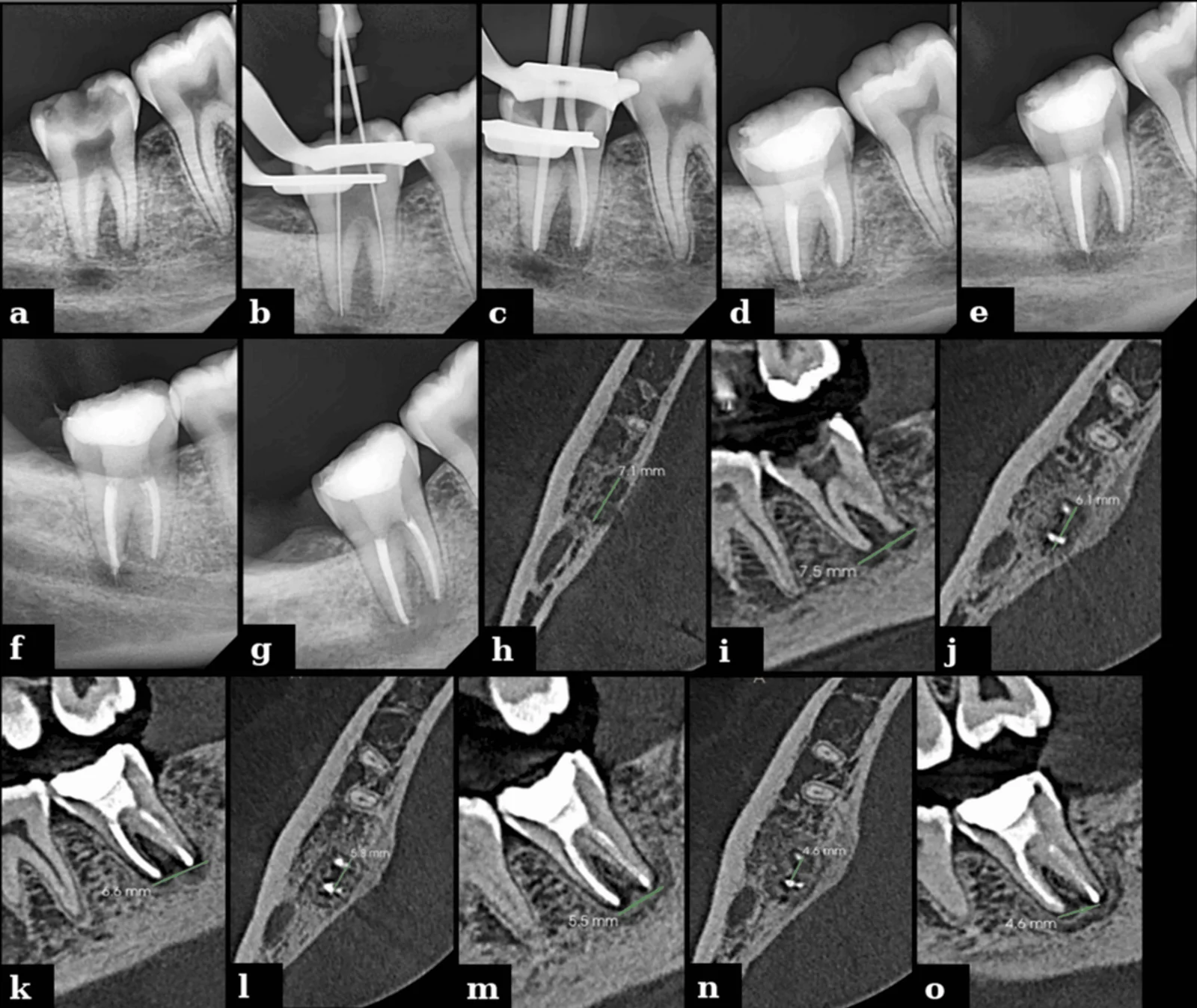
Serial radiographic evaluation of the osteoporosis group Two-dimensional RVG: (a) preoperative view, (b) working-length determination, (c) master cone, (d) obturation and postendodontic restoration, (e) one-month follow-up, (f) three-month follow-up, and (g) six-month follow-up. Three-dimensional CBCT, paired axial and sagittal views: (h,i) preoperative, (j,k) one-month follow-up, (l,m) three-month follow-up, and (n,o) six-month follow-up CBCT: cone-beam computed tomography; RVG: radiovisiography

**Figure 3:**
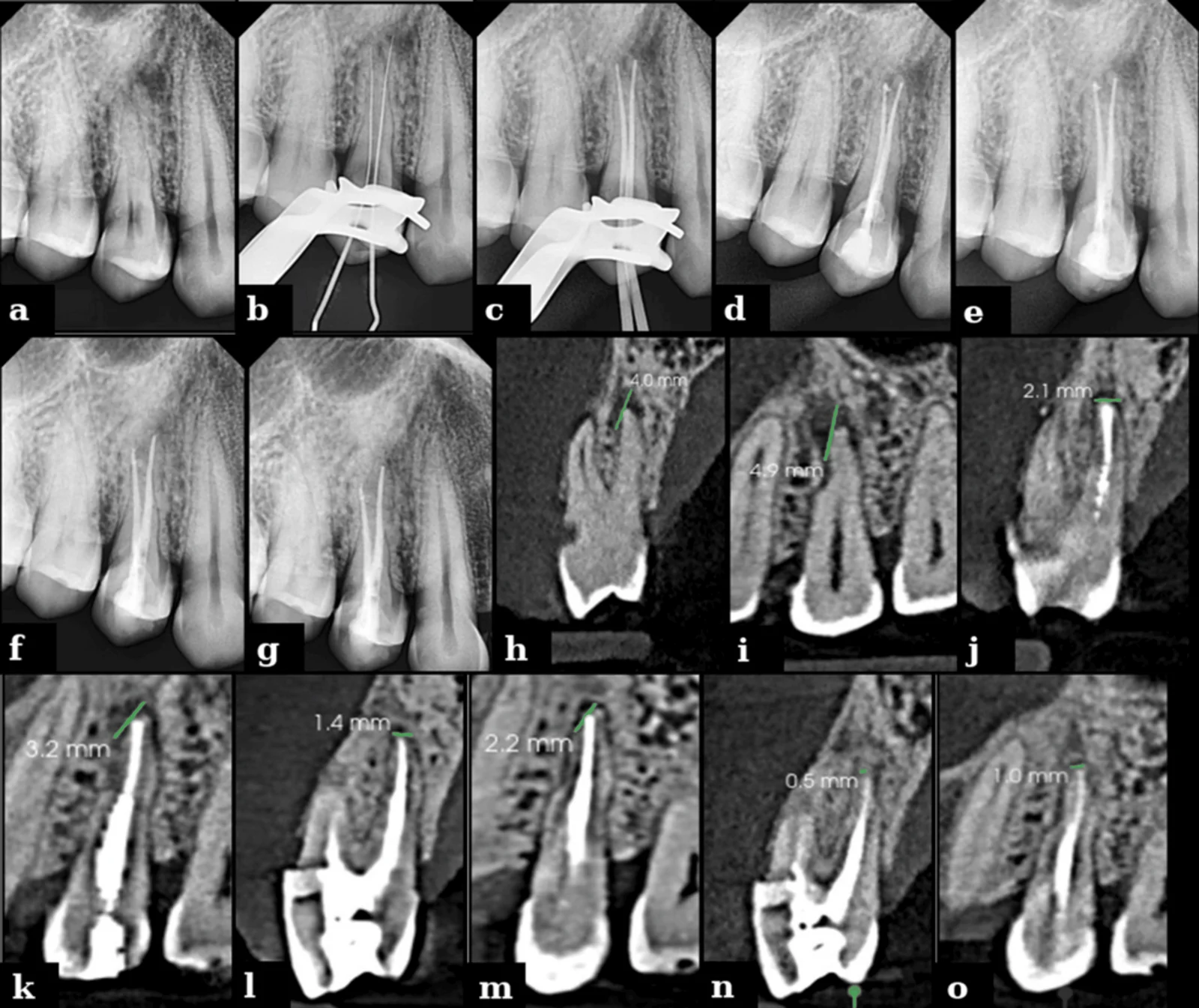
Serial radiographic evaluation of the rheumatoid arthritis group Two-dimensional RVG: (a) preoperative view, (b) working-length determination, (c) master cone, (d) obturation and postendodontic restoration, (e) one-month follow-up, (f) three-month follow-up, and (g) six-month follow-up. Three-dimensional CBCT, paired coronal and sagittal views: (h,i) preoperative, (j,k) one-month follow-up, (l,m) three-month follow-up, and (n,o) six-month follow-up CBCT: cone-beam computed tomography; RVG: radiovisiography

Statistical analysis

Data were entered into Microsoft Excel (Microsoft Corporation, Redmond, WA) and analyzed using Stata Statistical Software Release 17 (StataCorp LLC, College Station, TX). Continuous variables were reported as mean ± standard deviation or median (interquartile range), as appropriate, while categorical variables were presented as frequencies and percentages. Normality was assessed using the Shapiro-Wilk test. As most variables were nonnormally distributed, the Wilcoxon signed-rank test was employed for intragroup comparisons and the Kruskal-Wallis test for intergroup comparisons. A two-sided p value of <0.05 was considered statistically significant.

## Results

The study included 80 participants who met the eligibility criteria: 40 healthy controls, 20 patients with OP, and 20 patients with RA. Table 1 presents the baseline demographic and clinical characteristics of the participants. The mean age was highest in the RA group (55.1 ± 8.12 years), followed by the OP group (52.5 ± 8.62 years) and the control group (39.0 ± 12.72 years). Female participants constituted 61.3% of the study population, with the highest proportion observed in the OP group (80%). Mandibular posterior teeth were more frequently treated than maxillary posterior teeth (58.8% vs. 41.3%) (Table 1).

**Table 1:** Baseline demographic and clinical characteristics of study participants Sex distribution: Pearson's chi-squared test, χ²(2) = 4.27, p = 0.119 Maxillary vs. mandibular distribution: Pearson's chi-squared test, χ²(2) = 1.70, p = 0.427 df: degrees of freedom; SD: standard deviation; OP: osteoporosis; RA: rheumatoid arthritis

Group	Age (years), mean ± SD	Sex (male/female), n (%)	Maxillary teeth, n (%)	Mandibular teeth, n (%)	Total
Control	39.0 ± 12.72	19 (47.5)/21 (52.5)	19 (47.5)	21 (52.5)	40
OP	52.5 ± 8.62	4 (20.0)/16 (80.0)	6 (30.0)	14 (70.0)	20
RA	55.1 ± 8.12	8 (40.0)/12 (60.0)	8 (40.0)	12 (60.0)	20

Serial radiographic evaluation demonstrated a progressive reduction in periapical radiolucency measurements in all three groups throughout the six-month follow-up period. At baseline, the RA group exhibited the highest mean radiolucency (7.01 ± 3.18 mm), followed by the control (6.51 ± 3.00 mm) and OP groups (5.57 ± 2.31 mm). By six months, the control group demonstrated the lowest residual radiolucency (1.58 ± 1.18 mm) (Figure 1), whereas higher residual radiolucency persisted in the OP (2.60 ± 1.71 mm) (Figure 2) and RA (3.10 ± 2.44 mm) groups (Figure 3).

A significant reduction in PAI scores was observed within all groups between baseline and six months (Wilcoxon signed-rank test, p < 0.001) (Table 2). The mean PAI score decreased from 4.05 ± 0.846 to 1.82 ± 1.217 in the control group, from 4.20 ± 0.696 to 2.55 ± 1.276 in the OP group, and from 3.95 ± 0.686 to 2.60 ± 0.995 in the RA group.

**Table 2:** Intragroup comparison of baseline and six-month PAI scores in the study groups PAI: periapical index; SD: standard deviation; OP: osteoporosis; RA: rheumatoid arthritis

Group	Baseline PAI, mean ± SD	Six-month PAI, mean ± SD	Median change	p value
Control	4.05 ± 0.846	1.82 ± 1.217	4 → 2	<0.001
OP	4.20 ± 0.696	2.55 ± 1.276	4 → 3	<0.001
RA	3.95 ± 0.686	2.60 ± 0.995	4 → 3	<0.001

Intergroup comparison using the Kruskal-Wallis test showed no significant difference in baseline PAI scores among the three groups (p > 0.05). However, at the six-month follow-up, PAI scores differed significantly (p < 0.05), with the control group demonstrating lower residual PAI scores than the OP and RA groups.

## Discussion

This study aimed to assess the effect of OP and RA on NSET periapical healing using serial RVG and CBCT. All three groups showed significant improvement in radiography during the six-month follow-up period. However, OP and RA showed more residual periapical radiolucency and higher PAI scores at six months than the healthy controls, suggesting that periapical radiologic healing took longer in patients with these systemic diseases. CBCT-PAI scores were given based on Estrela et al. criteria [11], which offered a standardized method for assessing changes in periapical lesions.

The difference in healing noted between the OP and control groups may be due to changes in bone remodeling and mineral metabolism. OP is a decrease in bone mineral density, trabecular disruption, and a lack of balance in the process of osteoblastic bone formation and osteoclastic bone resorption, which can affect periapical bone regeneration. This relationship was also supported by the study conducted by Thanakun et al. [12] that showed a correlation between periapical lesion prevalence and changes in bone density in the mandible of postmenopausal women.

Other clinical research studies have confirmed an association between periapical conditions and OP [13,14]. Katz and Rotstein [15] reported increased prevalence of periapical lesions among patients with OP. Cadoni et al. [16] also assessed the periapical status of OP patients and presented results that agreed with the systemic bone status and periapical pathology. Kamali and Turkaydın [17], using age- and sex-matched controls, further investigated the endodontic and periapical status of patients with OP. These findings are similar to those of the present study, in which patients with OP showed a higher level of residual radiolucency and PAI score at six months when compared with healthy controls.

The RA group also showed less radiographic healing than the control group. Systemic inflammatory disease has been shown to be associated with apical periodontitis in the study of Karataş et al. [18]. Periapical conditions may therefore be influenced by systemic inflammatory disease. Later, Wu et al. [19] discovered common genes and biological pathways between RA and pulp and periapical disease using GWAS and transcriptome data. The results support the current clinical observation that standardized NSET was associated with more residual periapical changes in RA patients than in controls.

Experimental studies also suggest a role for systemic changes in bone metabolism in periapical healing. Qian et al. [20] showed that the follicle-stimulating hormone-induced effects were detrimental to bone loss in rats with experimental periapical periodontitis when the rats were ovariectomized. The results suggest that there may be a systemic component that results in increased osteoclastic activity and periapical bone destruction, and that this component is responsible for some of the slow healing seen in the OP group in the present study.

The association between OP and the course of apical periodontitis has been further examined clinically. Further evidence for the relevance of altered systemic bone status to periapical disease course was provided by Stanev and Vasileva [21] in their study on the effects of OP on the course of apical periodontitis. The relationship between the bone mineral status and periapical pathology was also supported by the report of López-López et al. [22], who reported that bone mineral density in postmenopausal women showed an association with the presence of radiolucent periapical lesions. Heuchert et al. [23] performed a systematic review and meta-analysis of mandibular radiomorphometric indices as indicators of decreased bone mineral density and OP. In summary, the findings support the hypothesis of an association between changes in systemic bone metabolism and periapical disease on a biological basis.

However, all three groups had significant improvement after NSET, although the rate of healing on the radiographs was different. This discovery revealed the relevance of the proper chemomechanical preparation, irrigation, obturation, and restoration in attaining endodontic success. All procedures in the current study were carried out by a single operator and applied to all patients with the same treatment protocol to minimize the treatment technique-related variation. This may thus be partly attributed to the systemic host factors observed.

The important strengths of this study were the prospective nature, the standardized endodontic treatment protocol, the inclusion of both OP and RA groups, and the RVG and CBCT serial evaluation. CBCT was used for three-dimensional evaluation of the periapical region, and the CBCT-PAI system was used for standardized evaluation of periapical changes [10]. However, the sample size, especially in the OP and RA groups, and the follow-up time of six months do not allow for long-term evaluation of periapical healing. Moreover, the study did not assess disease duration, disease severity, medication use, or systemic inflammatory status individually. All of these factors can affect bone metabolism and inflammatory response and could lead to differences in healing responses.

In conclusion, the results suggest that periapical outcomes after NSET in patients with OP and RA are highly promising, although healing may be slower than in systemically healthy individuals. The findings emphasize the possible significance of systemic bone and inflammatory diseases in evaluating endodontic healing.

Limitations

The present study was done with a relatively limited sample size, especially in the OP and RA groups, and a six-month follow-up period, which may not have been adequate to evaluate complete periapical healing. Additionally, severity of the disease, effect of medication, and systemic inflammatory status were not evaluated separately. Future studies involving larger cohorts, longer follow-up, and stratification based on disease characteristics are recommended. The present study evaluated OP and RA as predefined systemic exposure groups based on established diagnostic criteria rather than stratifying participants according to disease duration, disease activity, severity, or individual medication regimens. These variables may influence bone metabolism and inflammatory responses and could therefore contribute to variation in healing; however, assessment of their independent effects was beyond the primary objective of the present study. This limitation should be considered when interpreting the observed association between systemic disease status and periapical healing.

## Conclusions

With the limited nature of this study, periapical healing after NSET was delayed in OP and RA cases as compared to healthy cases. Although healing times were longer, marked radiographic improvement was seen in all groups, suggesting that successful periapical healing can be seen in medically compromised patients when adequate endodontic therapy is undertaken.
